# Novel Bionanocompounds: Outer Membrane Protein A and Laccase Co-Immobilized on Magnetite Nanoparticles for Produced Water Treatment

**DOI:** 10.3390/nano10112278

**Published:** 2020-11-17

**Authors:** Nathaly Rangel-Muñoz, Andres Fernando González-Barrios, Diego Pradilla, Johann F. Osma, Juan C. Cruz

**Affiliations:** 1 Department of Biomedical Engineering, Universidad de Los Andes, Carrera 1 este No 19A-40, Bogotá 111711, Colombia; in.rangel@uniandes.edu.co; 2 Grupo de Diseño de Productos y Procesos (GDPP), Department of Chemical and Food Engineering, Universidad de los Andes, Carrera. 1 este No. 19a–40, Bogotá 111711, Colombia; andgonza@uniandes.edu.co (A.F.G.-B.); d-pradil@uniandes.edu.co (D.P.); 3 CMUA, Department of Electrical and Electronic Engineering, Universidad de Los Andes, Carrera. 1 este No. 19a–40, Bogotá 111711, Colombia; jf.osma43@uniandes.edu.co; 4 School of Chemical Engineering and Advanced Materials, The University of Adelaide, Adelaide 5005, Australia

**Keywords:** co-immobilization, demulsification and treatment, heavy crude oil, O/W emulsions, OmpA, Laccase, magnetite nanoparticles

## Abstract

The oil and gas industry generates large amounts of oil-derived effluents such as Heavy Crude Oil (HCO) in water (W) emulsions, which pose a significant remediation and recovery challenge due to their high stability and the presence of environmentally concerning compounds. Nanomaterials emerge as a suitable alternative for the recovery of such effluents, as they can separate them under mild conditions. Additionally, different biomolecules with bioremediation and interfacial capabilities have been explored to functionalize such nanomaterials to improve their performance even further. Here, we put forward the notion of combining these technologies for the simultaneous separation and treatment of O/W effluent emulsions by a novel co-immobilization approach where both OmpA (a biosurfactant) and Laccase (a remediation enzyme) were effectively immobilized on polyether amine (PEA)-modified magnetite nanoparticles (MNPs). The obtained bionanocompounds (i.e., MNP-PEA-OmpA, MNP-PEA-Laccase, and MNP-PEA-OmpA-Laccase) were successfully characterized via DLS, XRD, TEM, TGA, and FTIR. The demulsification of O/W emulsions was achieved by MNP-PEA-OmpA and MNP-PEA-OmpA-Laccase at 5000 ppm. This effect was further improved by applying an external magnetic field to approach HCO removal efficiencies of 81% and 88%, respectively. The degradation efficiencies with these two bionanocompounds reached levels of between 5% and 50% for the present compounds. Taken together, our results indicate that the developed nanoplatform holds significant promise for the efficient treatment of emulsified effluents from the oil and gas industry.

## 1. Introduction

Research and development involving nanoparticles (NPs) has gained considerable attention during the last decade due to the wide spectrum of possible applications. NPs have unique properties, including nanometric size (which provides them with large surface areas), chemical stability in different environments, ease of synthesis, and low production costs [1]. NPs have been implemented for applications in a number industries including pharma [2], nutraceutical [3], food [4], agro-industrial [5], and cosmetics [6]. Moreover, they have been successfully explored as supports for the immobilization of various molecules of biological origin with the purpose of increasing their functionality and incorporating additional properties such as responsiveness to external stimuli; enhanced thermal, conformational, and long-term stability; and ease of transport and manipulation [7,8,9,10,11]. A group of molecules that has been successfully immobilized on NPs is proteins and peptides. Upon immobilization, these molecules extend their lifetimes, enhance their recyclability, and can even improve their biological function in non-native environments. This significant improvement ultimately leads to higher operation efficiencies and yields [11]. Moreover, bionanocompounds based on proteins and peptides immobilized on NPs have enabled a large body of applications in industrial wastewater treatment [9,12,13].

The oil and gas industry has been traditionally perceived as environmentally harmful. As a result of extraction and production activities, petroleum production generates large volumes of harmful effluents [12]. An important fraction of the effluents encompassing produced water are in the form of residual Oil-in-Water emulsions (O/W) [13]. These emulsions are difficult to treat and dispose of adequately. Consequently, significant effort has been devoted to the development of methods for the recovery of natural water sources and the remediation of wastewaters resulting from oil production activities [14,15,16,17]. Emulsions are a special type of colloids where a liquid is dispersed in the form of droplets (dispersed phase) in a second immiscible liquid (continuous phase) with a different polarity [13]. O/W emulsion preparations have been recently developed for the petroleum industry as pipeline aids for the transport of crude oil—e.g., the commercially available product Orimulsion^®^ (BITOR, Salager et al., 2001) [18,19]. The high stability of these systems generates a number of difficulties for their removal and high operating costs mainly due to the required extreme operating temperature conditions and relatively low efficiencies [20].

Traditional methods for emulsified wastewater treatment are based on mechanical (ultrafiltration) [21], physical-chemical (photocatalysis) [22], or biological (bioremediation) approaches [23]. The last group of processes has attracted significant attention and mainly refers to the incorporation of active biomolecules or whole microorganisms into different stages during the treatment of the recovered effluents [24]. This approach is advantageous due the low toxicity and environmental friendliness of these agents [25]. One of the most useful groups of biomolecules for emulsion bioremediation (due to their interfacial activity) are biosurfactants, which have been categorized into different families including lipids, proteins, and bacteria [24,25,26,27,28]. Despite the possible benefits, biosurfactants tend to face stability issues that compromise their performance, especially when subjected to operating conditions far from those observed in their native environments [29]. For this reason, their applicability has been largely limited to the food and biomedical industries, where the processing conditions are milder [26].

Proteins comprise a group of biosurfactants of great interest due to their superior performance [28]. Most of these have been derived from bacteria and are usually present in their membranes [27,30,31]. Outer Membrane Protein A (OmpA) is an example of a successful biosurfactant, which is a the transmembranal protein of *Escherichia coli* [28,32]. The surface activity of OmpA has been attributed to its amphiphilic structure, composed of two main hydrophilic and hydrophobic chains. The N-terminal (amino acids 1 to 171) is hydrophobic and consists of eight beta chains with six binding sites where organic compounds can interact. Meanwhile, the C-terminal (amino acids 180 to 325) is hydrophilic and located in the periplasm; therein, it binds to the peptidoglycan, thereby connecting it to the outer membrane [33]. OmpA was successfully tested as an amphipathic demulsifier in model dodecane–water emulsions; consequently, the potential use of OmpA for the treatment of refinery effluents has attracted significant interest [34,35,36]. The demulsification processes of crude oil emulsions have also been enabled by nanomaterials, as they have facilitated HCO removal [37]. This is attributed to their high surface/volume ratio, which is thought to be responsible for high interfacial activity [17,38]. Some of the most successful nanomaterials include graphene oxide nanoparticles [38,39], carbon nanotubes [15], and magnetite nanoparticles (MNP) [14,40]. For instance, MNPs have been employed to destabilize oil emulsions upon the application of magnetic fields [14,15,16,41,42].

In addition to difficult separation, HCO emulsions contain several recalcitrant compounds (i.e., phenols, acyclic acids, naphthenic acids, or saturated and aromatic carboxylic acids) that need to undergo chemical transformation to decrease their potential environmental impact [43,44]. The required transformations are traditionally conducted with the aid of ozonation and oxidation processes [45] or photocatalysis [46]. However, the use of microorganisms such as bacteria, yeasts, or algae for the degradation of those pollutants has been also considered an attractive route [25,47,48]. Alternatively, enzymes are considered efficient and selective catalysts for bioremediation processes, where they have been incorporated as highly active and selective agents for the transformation of the recalcitrant compounds [49,50]. One of the most effective families of enzymes for bioremediation purposes are Laccases, which belong to the multicopper oxidases and can catalyze the oxidation of various aromatic substrates. Accordingly, they have the ability to degrade compounds such as phenols, amines, and hydrolyzed poly-acrylamides [50,51,52,53]. 

An emerging idea for the comprehensive treatment of emulsified effluents is to combine some of the technologies discussed above. Thus far, such exploration has been only conducted for the bioremediation of the pollutants and particularly through enzymatic systems. For instance, Yi Hu et al. discussed combining Laccase and ABTS onto chitosan nanoparticles for 2,4-DCP removal. Similarly, Bernabé L. Rivas et al. tested the synergy between *Agaricus bisporus* and *Trametes versicolor* Laccases in diazinon degradation [54,55]. Here, we introduced the notion that superior results in separation and treatment can be achieved by combining the demulsifying properties of OmpA, the destabilizing and recoverability power of MNPs, and the catalytic activity of the Laccase enzyme. An avenue to accomplish this is through the co-immobilization of OmpA and Laccase molecules on MNPs. Nevertheless, several parameters need to be considered to assure that the proteins preserve their activity after immobilization. These include the surface chemistry of the support, enzyme packing density, enzyme charge, and isoelectric point [10]. Susceptibility to undergoing detrimental conformational changes upon contact with surfaces has been controlled by a number of strategies, including adsorption, covalent bonding, encapsulation, and the use of polymeric spacers (cross linking) [10,56]. Hydrophilic polymers have been successfully implemented for the covalent immobilization of several proteins [57]. One example of this is the polymers from the polyether amine (PEA) family, which provide flexibility and a local microenvironment that prevents conformational changes. The end result is a relatively large number of functional protein molecules on the surface of the material [57,58].

This work is therefore dedicated to investigating whether, by co-immobilizing OmpA and a Laccase on MNPs, it is possible to separate O/W emulsions and reduce some of the environmentally harmful compounds present in them. We immobilized biomolecules with the aid of a PEA surface spacer to maintain high levels of functionality for both proteins—i.e., interfacial activity (OmpA) and catalytic competency (Laccase). First, the obtained bionanocompounds were chemically characterized with the aid of X-ray diffraction and FTIR spectroscopy. The physical properties were determined via DLS, TEM, and TGA. Finally, the immobilization yield was indirectly quantified with an ABTS oxidation assay for the Laccase molecules. The demulsifying effect of free MNPs, OmpA, and MNP-PEA-OmpA and MNP-PEA-OmpA-Laccase bionanocompounds was tested in a synthetic O/W emulsion in the absence and presence of a magnetic field. Additionally, we identified an increase in the removal percentage of harmful compounds such as asphaltenes, alkenes, and aromatic molecules after treatment with the MNP-PEA-OmpA-Laccase bionanocompound.

## 2. Materials and Methods 

### 2.1. Materials

Iron (II) chloride tetrahydrate 98% (PanReac Quimica, SLU, Barcelona, Spain.) and Iron (III) chloride tetrahydrate (Merck, Munich, Germany), 98% sodium hydroxide (NaOH), dimethylformamide (DMF) (PanReac Quimica, SLU, Barcelona, Spain), 25% tetramethylammonium hydroxide (TMAH), 98% (3-Aminopropyl) triethoxysilane (APTES), 98% N-hydroxy succinimide (NHS), and 98% N-[3-dimethylammino)-propyl]-N’-ethyl carbodiimide hydro- chloride (EDC) (Merck, Darmstadt, Germany) were used for MNP synthesis and functionalization. Tryptone, yeast extract, sodium chloride (NaCl) 98%, lactose, and sodic phosphate (Na_2_HPO_4_) were all purchased from Merck KGaA, (Darmstadt, Germany). Protein purification required the Profinity^TM^ IMAC Resin (Bio-Rad Laboratories, Hercules, CA, USA) and buffers with sodium acetate (C_2_H_3_NaO_2_), 99.9% acetic acid (99.8%) (CH_3_COOH) (PanReac Quimica, SLU, Barcelona, Spain.), 85% phosphoric acid (H_3_PO_4_), imidazole (99%), nickel (II) sulfate 99.99% (NiSO_4_) (Merck, Darmstadt, Germany), and EDTA 99% (PanReac AppliChem, Chicago, IL, USA). Jeffamine M-600 polyetheramine (PEA) (Huntsman Corporation, Woodland, TX, USA) was used as a surface spacer and oxidized with the aid of KMnO4 (Sigma-Aldrich, Munich, Germany) to render free terminal carboxyl groups for further conjugation. Laccase from *Trametes versicolor* 0.5 U/mg, HCl, KMnO4, Tween 20, sodium bicarbonate 99.7% (NaHCO_3_), 98% magnesium chloride, and TritonX were purchased from Sigma-Aldrich, Munich, Germany. The 97% calcium chloride (CaCl_2_) was from PanReac Quimica, SLU, Barcelona, Spain; the ultrapure petroleum ether was Merck KGaA, (Darmstadt, Germany); and the nonylphenol ethoxylate 10 mol (TERGITOL™ NP.10 Surfactant) was from Dow Chemical, Midland, MI, USA. Heavy crude oil (CO) from a Colombian oilfield was used as reported by *Acosta* et al. [59]. 

### 2.2. Protein Production

Outer membrane protein A (OmpA) was recombinantly expressed in *Escherichia coli* K-12 W3110/pCA24N OmpA^+^. Bacteria were cultured overnight at 37 °C in Lurta-Bertani (LB) agar plates containing yeast extract (5 g/L), tryptone (10 g/L), NaCl (10 g/L), and chloramphenicol (50 μg/mL). Next, a colony was inoculated in LB medium (25% NaCl, 25% tryptone, 12.5% yeast extract, and 100 μg/mL of chloramphenicol) and incubated for 16 h at 37 °C and 250 RPM. Subsequently, fresh LB medium (5 L) in a Bioflo/Celligen 115 Bioreactor (New Brunswick Scientific, Edison, NJ, USA) was inoculated with the previous culture and maintained for about 2 h at 37 °C, 250 RPM, and a 2 L/min air flow. The culture was grown until reaching an optical density of 0.8 at 600 nm (OD600 nm). At this point, the medium was fed with isopropylthio-β-galactoside (IPTG) (2 mM) to induce expression. IPTG exposure was performed for 5 h. Bacteria were then collected and concentrated with the aid of a 0.22 μm cassette membrane in a parallel plate filter (Pellicon^®^ 2 Filters and Holders, Merck Millipore Corporatio, Burlington, MA, USA). The collected cells with overexpressed OmpA were lysed with an ultra-sonic tip Vibra-cell VCX-750 (Sonics & materials Inc., Newton, CT, USA) after adding a lysis buffer (NaH_2_PO_4_, NaCl, Tween 20, TritonX). Sonication proceeded for 40 cycles (20 s on, 40 s off) at 37% amplitude in ice. The obtained lysate medium was centrifuged at 4500 RPM and 4 °C for 2 h and the supernatant was recovered. Purification was conducted by passing the supernatant through a purification column packed with Profinity™ IMAC Resins, following the manufacturer’s instructions. Purified fractions were collected, and the presence of OmpA in each one of them was verified with sodium dodecyl sulfate polyacrylamide gel electrophoresis (SDS-PAGE). Fractions showing a single band at 35 kDa were lyophilized to preserve protein structure and stored at 4 °C until further use. 

### 2.3. Protein Characterization

OmpA protein was lyophilized for 48 h at −80 °C. The final concentration of the protein was quantified by colorimetric protein determination techniques using a commercial BCA Protein Assay Kit (Sigma-Aldrich, Munich, Germany) (Supplementary Figure S1). The secondary structure of OmpA was analyzed via second derivative methods [60] of its characteristic FTIR spectrum, as collected with a Bruker Alpha II FTIR Eco-ART (Bruker Optik GmbH, Ettlingen, Germany). Spectra were measured in the range of 4000–400 cm^−1^ with a spectral resolution of 2 cm^−1^ at room temperature.

### 2.4. Polyetheramine (PEA) Oxidation

A total of 3 g of Jeffamine M-600 polyetherimine (PEA) was oxidized in 50 mL of a 0.015 M solution of KMnO_4_ following the protocol of Feng et al. [61]. The mixture was kept under magnetic stirring for 12 h at room temperature. The MnO_2_ precipitate resulting as byproduct of the oxidation reaction was filter under vacuum with a 0.7 µm paper filter until obtaining a transparent solution. The pH of the sample was adjusted to 2.0 by adding 10 M of hydrochloric acid (HCl) dropwise. Excess KMnO4 and HCl was removed by dialysis using a 100–500 Da cut-off molecular weight membrane with ultra-pure water for 12 h. Finally, the water excess in the PEA solution was removed in a rotary evaporator (Vap Value Digital Vertical Heidolph, Schwabach, Germany) under vacuum at 80 °C for 6 h. The PEA oxidation was verified by FTIR, and its approximate concentration was determined by the chemical titration of the carboxyl groups (acid value). 

### 2.5. Synthesis of Magnetite Nanoparticles

Magnetite nanoparticles were synthesized by the co-precipitation method. Briefly, 2.528 g of FeCl_2_.4H_2_O (2.73 mmol) and 7.56 g of FeCl_3_.6H_2_O (27.96 mmol) were dissolved separately in 40 mL of ultra-pure water to obtain 0.3 M and 0.7 M solutions, respectively. Additionally, 5.6 g of NaOH were dissolved in 40 mL of water. Chlorides solutions were then mixed in the presence of 40 mL of ultra-pure water and 2 mL of 2% (*v*/*v*) solution of TMAH (to avoid agglomeration), followed by heating up to 90 °C. Finally, the NaOH solution was manually dropped into the chloride mixture at a rate of 3 mL/min while maintaining vigorous agitation at 1500 RPM and the temperature at 90 °C. The reaction was carried out for 1 h. The obtained MNPs were washed at least 3 times with ultra-pure water with the aid of a strong permanent magnet for rapid separation and sonicated in an ultrasonic bath (2800 Cleaner, Branson Ultrasonics, Brookfield, CT, USA) in between washes.

### 2.6. MNP Characterizations

The size of the obtained MNP was measured using a Dynamic Light Scattering (DLS) instrument (Zeta-Sizer Nano-ZS, Malvern Instruments, Malvern, UK) and Transmission Electron Microscopy (TEM) (Tecnai F20 Super Twin TMP, ThermoFisher, MA, USA). Additionally, the purity of the crystalline phase of magnetite was verified via X-Ray Diffraction (XRD). Finally, effective synthesis was confirmed by Fourier-transform infrared spectroscopy (FT-IR) and thermogravimetric analysis (TGA) (TA Instruments, New Castle, DE, USA). Infrared spectra were recorded using an A250/D FT-IR (Bruker, Billica, MA, USA). The FT-IR parameters were specified with the aid of the OPUS Spectroscopy Software (Bruker, Billica, MA, USA). Spectra were recorded in the range of 4000–400 cm^−1^ with a spectral resolution of 2 cm^−1^.

### 2.7. OmpA and Laccase Immobilization: Magnetite-PEA-OmpA-Laccase Bionanocompounds 

The silanization of MNP was carried out with an initial sample of 100 mg of MNP in 30 mL of ultra-pure water. The process was started by sonicating the MNP for 40 min to avoid agglomeration. Next, 200 mL of a 2.5% (*v*/*v*) solution of TMAH was added to help resuspending the MNP. Then, 20 µL of 99% glacial acetic acid was added prior to the conjugation of APTES (200 µL). This compound rendered free amine groups on the surface of the MNPs. The reaction was carried out at 60 °C and 250 RPM for 1 h. Silanized nanoparticles were washed five times with ultra-pure water and 1.5% (*w*/*v*) NaCl solution to remove excess APTES, aided by a strong permanent magnet.

Silanized MNP was resuspended in 30 mL of ultra-pure water and sonicated for 40 min. Then, 2 mL of 2% (*v*/*v*) of glutaraldehyde (GA) was added to the MNP sample and left under mechanical agitation at 250 RPM and room temperature for 24 h. Oxidized PEA was then conjugated to the obtained MNP-AP-GA by adding 1 mL of PEA solution in a 1:2 molar ratio with respect to the free amine groups. The reaction proceeded overnight at 250 RPM and room temperature. Finally, the PEA-coated magnetite nanoparticles were washed three times with ultra-pure water and three times with 1.5% (*w*/*v*) NaCl solution.

To obtain the MNP-PEA-OmpA-Laccase bionanocompounds, OmpA and Laccase were conjugated according to the calculations presented in Supplementary Data SI2. Both OmpA and Laccase were sequentially immobilized at a 1:1 molar ratio. Accordingly, the required amount of protein was suspended in 2 mL of ultra-pure water with 5 mg of NHS and 5 mg of EDC. The proteins were conjugated to the carboxyl groups of oxidized PEA (1 equivalent) by their N-terminal to form amide bonds. After adding proteins, the reaction was conducted for 24 h at 250 RPM and room temperature. Next, the samples were thoroughly washed with type I water (ultra-pure water with a resistivity >18 MΩ-cm and conductivity of <0.056 µS/cm) and the aid of a strong permanent magnet to remove excess reagents.

### 2.8. Characterization of Magnetite-PEA-OmpA-Laccase Bionanocompounds

Prior to characterization, an amount of each sample was lyophilized and subsequently characterized via FTIR and TGA. The obtained MNP-PEA-OmpA, MNP-PEA-Laccase, and MNP-PEA-OmpA-Laccase bionanocompounds were evaluated via Fourier Transform Infrared Spectroscopy (FTIR) by a Bruker Alpha II FTIR Eco-ATR (Bruker, Billica, MA, USA). Spectra were collected in the range of 4000–400 cm^−1^ with a spectral resolution of 2 cm^−1^. The second derivative of magnetite-PEA-OmpA-Laccase bionanocompounds was calculated with the OriginPro8 Software and subsequently employed to determine alterations in the secondary structure of proteins after immobilization [2,60]. TGA was performed to estimate the thermal stability of magnetite nanoparticles and bionanocompounds. The experiment was carried out with 5–10 mg of lyophilized sample, and the percentage of mass loss was recorded as a function of temperature from 10 to 800 °C at a heating rate 10 °C/min under a nitrogen atmosphere. 

### 2.9. Crude Oil in Water (O/W) Emulsion Preparation

The O/W emulsion formulation was based on previous studies by Aburto et al. [17,19]. The formulated emulsion contained 1 wt% of crude oil (dispersed phase) in water (continuous phase). Nonylphenol ethoxylate at 10 mol (NPE10) with HLB 13.2 was used as a surfactant to achieve a stable direct emulsion, as described by Loh et al. [62]. For stabilization, the continuous phase was prepared as a brine solution following the procedure described in Øye et al. [63,64]. The composition of the prepared brine is shown in Table 1. 

Stable emulsions were prepared as follows: (i) A volume of brine solution, corresponding to 97% of the total volume of the sample, was transferred to a vessel and heated to 60 °C in a hot plate. This temperature was kept constant throughout the entire experiment. (ii) In a separate hot plate, 0.6 mL of heavy crude oil was transferred to a vessel and heated to 60 °C. A homogenizer was then connected to the hot brine container, which was equipped with a propeller located such that a ratio of propeller (Da) to vessel diameter (Dt) of about 1 (i.e., Da/Dt ≈ 1) was maintained (see Supplementary Figure S2). (iii) A volume of NP-10mol (NP) surfactant was pipetted into the hot brine container with direct displacement to a total volume corresponding to 3% of the sample. (iv) The mixture was then homogenized at an initial speed of 2000 RPM to incorporate the surfactant while avoiding foam formation. (v) The pre-heated hot crude oil, corresponding to 1 wt% of the sample, was pipetted into the brine at a rate of 100 mL/s by locating the tip close to the propeller. The agitation speed was then increased to 5000 RPM for 15 min at 60 °C.

### 2.10. Physical Stability of O/W Emulsions

Bottle testing is a widely used protocol for the easy assessment of demulsifier performance in oilfields. The incorporation of demulsifier at different concentrations is required, considering the significant amount of water added to homogenize it. The mixture was then added to the dispersed phase. Traditional bottle tests were carried out to determine the stability of the O/W emulsion. The stability of HCO in water (O/W) emulsions was measured by following the changes in transmission signals and backscattering of light over time. This was also useful to assure the reproducibility of the emulsion manufacturing protocol. The Δ backscattering (ΔBS) measurement and the Turbiscan Stability Index (TSI) were recorded in a Turbiscan Lab Stability Analyzer (Formulaction SA, Toulouse, France) at conditions of transmission (0° from the incident light beam) and backscattering (135° from the incident light beam) by utilizing a pulsed near-infrared light source (λ = 850 nm). For this purpose, samples were carefully transferred to a glass vial by preventing bubble formation at the top of the container. Measurements took place every minute for 1 h at room temperature (25 °C). HCO drops were imaged in an optical microscope with a 100X objective (NA = 40). Image analysis was performed in the open access software Image J^®^ (https://imagej.nih.gov/ij/). 

### 2.11. Demulsification Effect of MNP and OmpA in O/W Emulsion and Bottle Test

The destabilizing effect of MNP and OmpA on the formulated O/W emulsion was evaluated separately in the Turbiscan for 1 h at room temperature. For this purpose, 16 mL of the O/W emulsion were placed in a glass vial along with 200, 500, 1000, or 5000 ppm (i.e., 3.2, 8, 16, 80 mg or 500, 1000, 5000 µg/mL) of MNP or OmpA. The mixture was then homogenized at 100 RPM. The ΔBS was monitored to determine the range of bionanocompound concentrations required for effective HCO removal. 

### 2.12. O/W Emulsion Separation and Treatment 

An effective concentration of 5000 ppm (5000 µg/mL) of OmpA and MNP for HCO removal was determined. Concentrations of bionanocompounds above 5000 ppm failed to produce a further improvement in separation (data not shown). Such optimal concentration corresponded to an effective treatment ratio of 1:5 (i.e., 1 mL of HCO per 5 mg of MNP-PEA-OmpA or MNP-PEA-OmpA-Laccase bionanocompound). Consequently, the demulsification tests with MNP-PEA-OmpA and MNP-PEA-OmpA-Laccase bionanocompounds were conducted at this concentration. After the incorporation of the bionanocompound, the sample was left at rest for 1 h to monitor separation by direct observation. Next, the bionanocompounds were recovered by placing a neodymium permanent magnet (1.2 T) at the bottom of the glass vial. Even though the bionanocompounds were easily recovered after treatment, further studies will be required to assess whether they could be recycled into the process. Subsequently, the remaining HCO in the sample was extracted with petroleum ether at a 1:3 mass ratio (sample:ether) in an ultrasound bath for 5 min. The non-polar phase with the HCO dissolved in ether was collected and analyzed via UV-VIS spectrophotometer (GENESYS^TM^ 10S, Thermo Fisher Scientific ^TM^, Waltham, MA, USA) at 225 nm, where the HCO exhibits a characteristic peak [15]. The maximum content of HCO was recorded as relative percent by taking as reference samples in the absence of bionanocompounds (i.e., 100% HCO content). 

Finally, the bioremediation capacity of the MNP-PEA-OmpA/Laccase was evaluated via Gas Chromatography coupled to Mass Spectrometry (GC-MS) in an Agilent technologies (Santa Clara, CA, USA) 6890N gas chromatography instrument coupled to a selective mass detector (MSD) 5975 in Full Scan mode. The GC was equipped with a HP-5MS column (30 m × 0.325 m × 0.25 μm) operating at a 1.2 mL/min flow rate. The initial oven temperature was held at 35 °C for 5 min. It was then increased at a rate of 8 °C/min to 220 °C and kept there for 5 min for a total detection time of 33.125 min. The injection mode was pulsed splitless at a 10 mL/min flow rate of Helium as entrainment gas at 200 °C. The role of the Laccase was obtained by comparing the GC-MS results obtained with the full bionanocompound (i.e., MNP-PEA-OmpA-Laccase) with those obtained with the MNP-PEA-OmpA bionanocompound. Prior to injection into the GC-MS system, the sample was extracted with dichloromethane and concentrated to 1 mL with N_2_ flow. Quantitative analyses were conducted by measuring the relative content of molecules before and after treatment.

## 3. Results and Discussion

### 3.1. Proteins, PEA and MNPs Characterization

**Figure 1:**
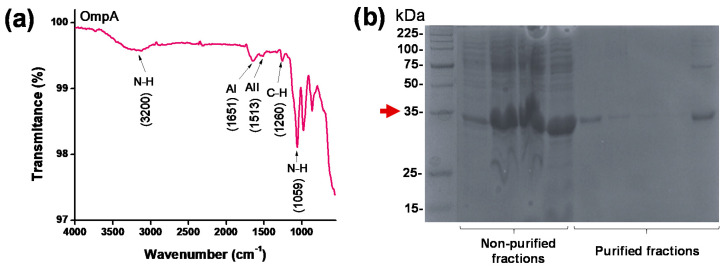
(**a**). FTIR spectrum of the dried OmpA protein. (**b**). Sodium dodecyl sulfate polyacrylamide gel electrophoresis (SDS-PAGE) of collected fractions during the purification procedure. Lane 1 shows the molecular weight marker while lanes 2 to 4 are for fractions collected of lysate supernatant and after washing. Lanes 5 to 10 are for fractions after elution from the Ni column. The band at about ~35 kDa corresponds to the OmpA.

The purity of the obtained protein was approximately 94%. Figure 1a shows the FTIR spectrum of dried OmpA. Peaks at 1651 cm^−1^ and 1513 cm^−1^ are associated with the Amide I and Amide II bands, respectively. The peak at 1260 cm^−1^ corresponds to the C-O stretching vibrations. The peaks at 1059 and 3200 cm^−1^ can be attributed to the N-H stretching bands vibration [65]. The secondary structural changes of OmpA were determined by the second derivative of Amide I band (Supplementary Figure S3). Peak assignment was according to previous reports and can be consulted elsewhere [2]. The Amide I was selected for this deconvolution because is the most sensitive spectral region of the protein which originates from the C=O stretching vibration of the amide group coupled with the in-phase bending of the N-H bond and stretching of the C-N bond [60]. Purified fractions of OmpA were confirmed in the SDS-PAGE gel, as shown in Figure 1b. The red arrow (~35 kDa) indicates the approximate molecular weight of OmpA, as reported previously [35]. Lanes 2–4 are the collected fractions after recovering the lysis supernatant and the first washing buffer, while lanes 5–10 are fractions collected after elution from the Ni column.

**Figure 2:**
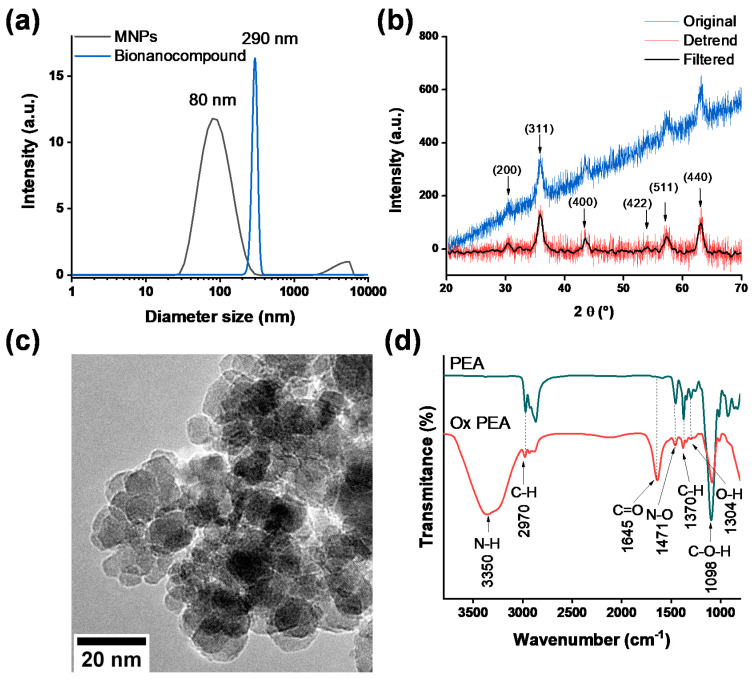
Magnetic nanoparticle (MNP) characterizations. (**a**) DLS histogram of the size distribution of MNP (gray) and MNP-PEA-OmpA/Laccase bionanocompound (blue). (**b**) XRD diffractogram of the dried MNPs. (**c**) TEM micrograph of MNPs. (**d**) FTIR spectra of PEA (green) and oxidized PEA (red).

Figure 2a shows the hydrodynamic diameter distribution of magnetite nanoparticles (MNP) as determined by DLS. The hydrodynamic diameter of bare MNPs approached an average of 80 nm with a polydispersity index (PI) of 22.4%. The average diameter of MNPs was used to calculate the maximum number of protein molecules that can be immobilized on the surface (Supplementary Materials, Section SI2). The XRD diffractogram of powder MNPs is shown in Figure 2b. To appropriately interpret the data, the obtained diffractogram was smoothed and filtered out by means of a Savitzky–Golay Filter [66]. Subsequently, peaks corresponding to the Bragg diffraction planes of the crystalline phase of Fe_3_O_4_ were identified (200, 311, 400, 422, 511, 400) and corroborated the absence of precursor impurities and unwanted crystalline phases such as Hematite [67]. TEM images show various aggregates with individual particles exhibiting an average diameter of ~30 nm (Figure 2c). Sizes of aggregates estimated from DLS and TEM measurements might not be comparable because in DLS, the sample is suspended in an aqueous medium, while for TEM the samples are dried out at room temperature before observation, which might lead to uncontrolled aggregation processes. Additionally, the drying process might induce the formation of salt crystals from excess reagents [68].

PEA oxidation was confirmed by Fourier transformed infrared spectroscopy (FTIR). Figure 2d shows the FTIR spectra of both PEA and oxidized PEA. Prior to oxidation, FTIR spectrum of PEA shows two absorption bands at around 1098 cm^−1^, which can be attributed to the C-O-H stretching vibration and an additional band at 1471 cm^−1^, which can be correlated with the C-H bending vibration. Compared to the PEA IR spectrum, oxidized PEA exhibits three different vibrational bands at about 1645, 1370, and 1304 cm^−1^, which can be explained by the presence of C=O stretching, C-H bending, and O-H stretching, respectively. Of interest are the C=O stretching bands at 1645 and 3350 cm^−1^, because they can be associated with the COOH bonds of carboxyl groups needed for the conjugation of OmpA and Laccase. The peak at 2867 cm^−1^ corresponds to the amorphous PEA (C-H asymmetric stretching) [61].

### 3.2. Bionanocompounds Characterization

**Scheme 1:**
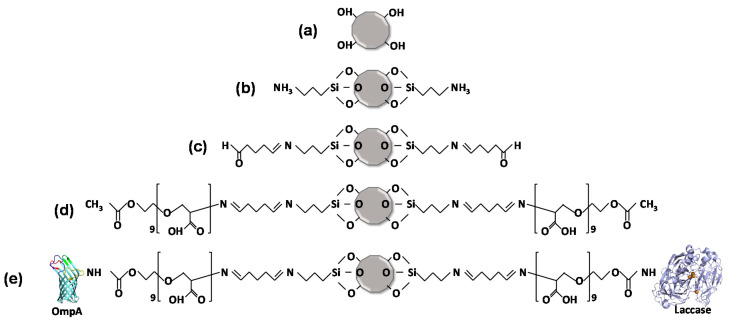
Chemical structure along the intermediate steps of the synthesis to arrive to the final bionanocompound. (**a**) Magnetite (Fe_3_O_4_) nanoparticle. (**b**) MNP-APTES functionalization. (**c**) MNP-APTES-Glutaraldehyde (GA). (**d**) MNP-APTES-GA-PEA nanocompound. (**e**) MNP-PEA-OmpA-Laccase bionanocompound.

**Figure 3:**
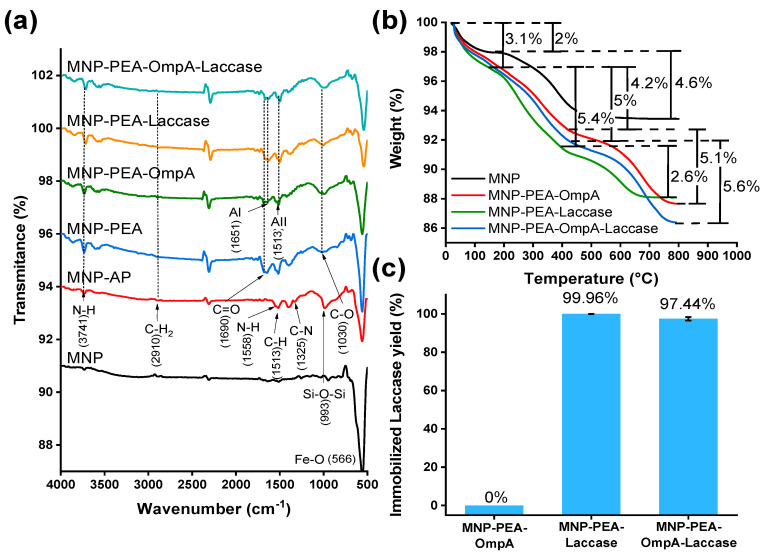
Characterizations of bionanocompounds: (**a**) FTIR spectra of MNP (black), silanized MNP (red), PEA coated MNP (blue), immobilized MNP-PEA-OmpA (green), MNP-Laccase (yellow) and MNP-PEA-OmpA-Laccase (turquoise). (**b**) Thermogravimetric assay (TGA) of MNP (black), MNP-PEA-OmpA (red), MNP-PEA-Laccase (green), and MNP-PEA-OmpA-Laccase (blue). (**c**) Immobilization yield (%) of immobilized Laccase in all samples, as calculated from an enzymatic assay based on the oxidation of ABTS.

The hydrodynamic diameter of the bionanocompound is reported in Figure 2a (blue). Contrary to the case of MNPs, after functionalization with OmpA and Laccase the average size approached 290 nm with a PI of 49%. The conjugation of OmpA and Laccase on PEA-modified MNP is schematically shown in Scheme 1e. Successful conjugation was confirmed by FTIR, TGA and an enzymatic activity assay. The FTIR spectra after each conjugation step are shown in Figure 3a. Bare magnetite (MNP) shows an absorption band at 566 cm^−1^, which can be attributed to the Fe–O bonds of iron oxide. This band continues to be present along all conjugation steps [61]. The presence of the Si-O-Si stretching vibration at about 993 cm^−1^, the bending vibrations of C–N and C–H at 1325 cm^−1^ and 1513 cm^−1^ as well as N–H bands at 1558 cm^−1^ and 3741 cm^−1^ confirm the silanization with APTES (MNP-AP). The conjugation of PEA on silanized magnetite (MNP-AP-PEA) was confirmed by the absorption peak at 1041 cm^−1^ for the C–H bending vibrations and at 1690 cm^−1^ related to the presence of C=O stretching in the backbone of the polymer. Finally, the conjugation of OmpA and Laccase on the surface of MNP (MNP-PEA-OmpA-Laccase) was verified by the presence of the amide I and II bands at 1651 and 1513 cm^−1^, respectively.

The secondary structural changes of immobilized OmpA were assessed with the aid of the second derivative of the FTIR spectra (Supplementary Figure S3). The results suggest that no significant secondary structural changes were observed for the protein after immobilization. The analyses were performed by direct comparison of the deconvoluted spectra prior and after immobilization [2]. In this regard, prior to immobilization vibrations corresponding to β-sheet are indicated at 1695, 1641, and 1625 cm^−1^. Additionally, a-helix structure and 3_10_-helix were identified at 1655 and 1664 cm^−1^, respectively [60]. After immobilization, the protein structure was conserved in their vibrations, as shown in Supporting Figure S3.

The conjugation efficiencies after each functionalization step were assessed with the aid of thermogravimetric analysis (TGA). TGA thermograms for bare magnetite (MNP) and the MNP-PEA-OmpA, MNP-PEA-Laccase, and MNP-PEA-OmpA-Laccase bionanocompounds are shown in Figure 3b. Magnetite exhibited a first weight loss of 2% at about 120 °C mainly due to dehydration. This was followed by a second weight loss of 4.6%, which can be attributed to physically adsorbed organic compound residues left by the synthesis and functionalization processes [15]. For the bionanocompounds, the first dehydration weight losses approached 4.2%, 5.4%, and 5% for MNP-PEA-OmpA, MNP-PEA-Laccase, and MNP-PEA-OmpA-Laccase, respectively. A second weight loss of 5.1% and 2.6% for MNP-PEA-OmpA (Red) and MNP-PEA-Laccase (green) indicated a similar immobilization efficiency for individual molecules. Co-immobilization (MNP-PEA-OmpA-Laccase) led to a second weight loss of 5.6%, which shows a subtle improvement in efficiency. This could be attributed to the interplay and competition of protein-protein and protein-surface interactions during immobilization [69,70,71,72]. Further studies will be required to maximize the efficiencies. 

### 3.3. Immobilization Yield Determination

As a well-known oxide-reductase, the enzymatic activity of Laccase can be easily determined by measuring the rate of oxidation of 2,2′-azino-bis(3-ethylbenzothiazoline-6-sulphonic acid) (ABTS) [73]. For this purpose, after immobilization the activity of excess laccase in the medium was measured colorimetrically by monitoring the conversion of ABTS with respect to a calibration curve. An estimate of immobilization yield was calculated according to Equation (1) by comparing the activity of initially added laccase with that of the laccase remaining after immobilization, where U remaining Laccase is the catalytic activity unit contained in the recovered immobilization medium and U added Laccase is the unit initially added for immobilization according to the calculations reported in Supplementary Materials, Section SI2 [70,72].

((U remaining Laccase)/(U added Laccase)) × 100.(1)

The enzyme contents were estimated based on the enzymatic activity as described in Supplementary Materials, Section SI5 and Figure S4. This approach followed previous reports where the number of active immobilized molecules has been estimated by quantifying their catalytic activity towards a substrate with the aid of colorimetric or fluorescent methods [69,74,75,76]. Figure 3c shows the immobilization yield obtained according to content of Laccase. For the negative control (i.e., MNP-PEA-OmpA), the immobilization yield is 0%. In contrast, MNP-PEA-Laccase and MNP-PEA-OmpA-Laccase bionanocompounds showed very high and similar immobilization yields of 99.96% and 97.44%, respectively. This provides further evidence for the successful completion of the co-immobilization process and that such process is equally effective for molecules immobilized independently or simultaneously (even if the process occurs sequentially). According to the obtained yields, the amount of Laccase in the MNP-Laccase bionanocompounds corresponds to 0.49 U/mg_MNP_ or 0.22 mg/mg_MNP_ (Supplementary Materials, Section SI2 and Table S1). Similarly, the amount of immobilized enzyme in the MNP-PEA-OmpA-Laccase bionanocompounds was 0.11 mg/mg_MNP_ or 0.23 U/mg_MNP_. According to the TGA and enzymatic assays, we assumed that the equivalents of immobilized proteins were about the same, which allowed us to calculate that the amount of OmpA in the MNP-PEA-OmpA (99.96% yield) was 0.12 mg/mg_MNP_, while for the MNP-PEA-OmpA-Laccase (97.44% yield) it was 0.06 mg/mg_MNP_.

One of the most important properties of MNPs is the interfacial activity, which can be further improved by functionalization with interfacially active molecules—e.g., amphipathic surfactants. Several other materials have been coated on the surface of MNP to improve their porosity, stability, or affinity for specific interfaces [77]. Examples of them include APTES [40], polymers [14,41], carbon structures [15,38,39], and enzymes [69,78]. The process of co-immobilization is intended to not only take advantage of the properties of MNPs, but also the properties of the interfaced molecules either independently or synergistically. This facilitates, for instance, one-step and one-pot strategies where just one system is possible to conduct multiple reactions. This approach is economically attractive as it considerably reduces the number of processing steps and consequently, the investment in equipment and involved personnel [69,79]. One major challenge is to assure the high immobilization efficiencies of active molecules. Here, such estimates for the proteins immobilized on the MNP-PEA-OmpA-Laccase bionanocompounds were somewhat problematic, considering that one of them (i.e., OmpA) shows no catalytic activity. Moreover, conventional methods for protein contents estimation such as BCA or Bradford are indifferent to the type of the protein and led to considerable error by the interference with the MNP support.

### 3.4. O/W Emulsion Stability and Characterization

**Figure 4:**
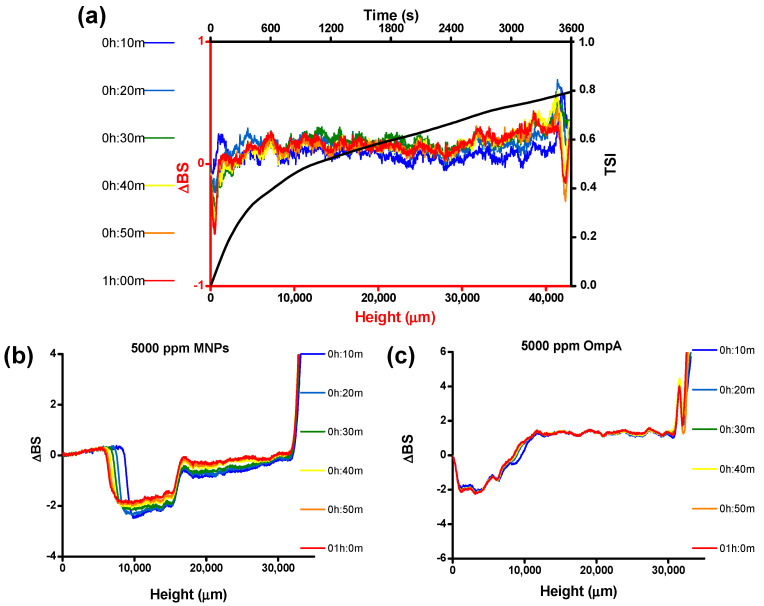
O/W emulsion characterization. (**a**) ΔBackscattering (ΔBS) for a representative O/W stabilized emulsion (red layer) and time evolution of the Turbiscan Stability Index (TSI) (black layer). Time evolution of ΔBackscattering (ΔBS) for O/W emulsion prepared with 5000 ppm of (**b**) bare MNP and (**c**) free OmpA. The ΔBS was monitored for 1 h at room temperature.

The physical stability of the prepared O/W emulsions was followed by monitoring the change in transmission signal and backscattering at room temperature in a Turbiscan Analyzer. This approach allowed quantification of destabilization phenomena such as sedimentation, creaming, aggregation, flocculation, and coalescence. The Turbiscan Stability Index (TSI) was also determined as a measure of the relative importance of the destabilization phenomena [80]. Low values of the TSI parameter usually indicate samples with a high stability. Figure 4a shows the ΔBackscattering (ΔBS) of the prepared emulsion after 1 h (red layer). Two more experiments confirmed the reproducibility of the collected data (not shown). No destabilization phenomena were observed within the experimental window. Figure 4a shows that the TSI parameter approached 0.8 after 1 h (black layer). This result is in line with the stability demonstrated by the ΔBS data.

### 3.5. Demulsification Effect of Free MNP and OmpA

OmpA and MNP were added to the emulsion at concentrations of 200, 500, 1000, and 5000 ppm and their destabilizing effect was recorded with the aid of a Turbiscan instrument. We explored increased concentrations of demulsifier to analyze whether destabilizing phenomena might occur at a window of higher concentrations. The ΔBS results for O/W emulsions in the presence of OmpA and MNP are shown in Supplementary Figures S5 and S6, respectively. According to our results, at protein concentrations from 200 to 1000 ppm, changes in the stability of the emulsion were negligible; however, at 5000 ppm of both MNPs (Figure 4b) and OmpA (Figure 4c) significant changes were observable. As evidenced by the important changes in the ΔBS signal at the bottom of container in Figure 4c, we hypothesize that at the beginning of the experiment insoluble protein clusters tend to precipitate and induce the sedimentation of some of the components of the emulsion. The decrease in ΔBS signals at the top of the container indicates the presence of bubbles. In contrast, the ΔBS signal profiles for destabilization in the presence of MNPs (Figure 4b) indicate creaming at the top of the container most likely due to the movement of oil drops to this region. Moreover, the ΔBS decrease at the center of the container indicates clarification of the sample while the increase at the bottom is for the sedimented MNPs covered with HCO [81]. Interestingly, a closer inspection of the ΔBS profiles at 500 ppm MNP revealed a possible stabilizing effect. This was evidenced by profiles with even smoother tendencies than those observed for the blank (Figure 4a, red layer).

This agrees well with previous studies where superior stabilization is achieved by mixing nanoparticles with chemical surfactants in systems known as Pickering emulsions [82,83]. This stabilization phenomenon usually takes place when the particles exhibit sizes close to those of the emulsion droplets—i.e., ~100 nm [82]. The particles can also be formed in situ by the presence of molecules such as proteins and starch [37,84]. MNPs have attracted considerable attention to alter the stability of emulsions and dispersions due to their high interfacial activity and the possibility to act in a controlled and reversible manner [83]. Recent reports showed that Pickering emulsion stabilization has been also achieved by the presence of MNPs of ~100 nm at concentrations ranging from 0.1 to 5 wt% [40,83].

### 3.6. Removal Efficiency

We also tested MNP-PEA-OmpA and MNP-PEA-OmpA-Laccase for demulsification and estimated their remediation capabilities by looking at the changes in chemical composition via GC-MS. Under this perspective, we conducted bottle tests with MNP-PEA-OmpA and MNP-PEA-OmpA-Laccase bionanocompounds at a concentration of 5000 ppm. This corresponds to an approximate concentration of 600 ppm of OmpA for the MNP-PEA-OmpA bionanocompound. In the case of the MNP-PEA-OmpA-Laccase bionanocompound, the corresponding concentrations were 300 ppm for OmpA and 550 ppm for Laccase. Finally, by assuming that all Laccase molecules remain active after immobilization, we calculated a catalytic activity of 18.4 U. 

**Figure 5:**
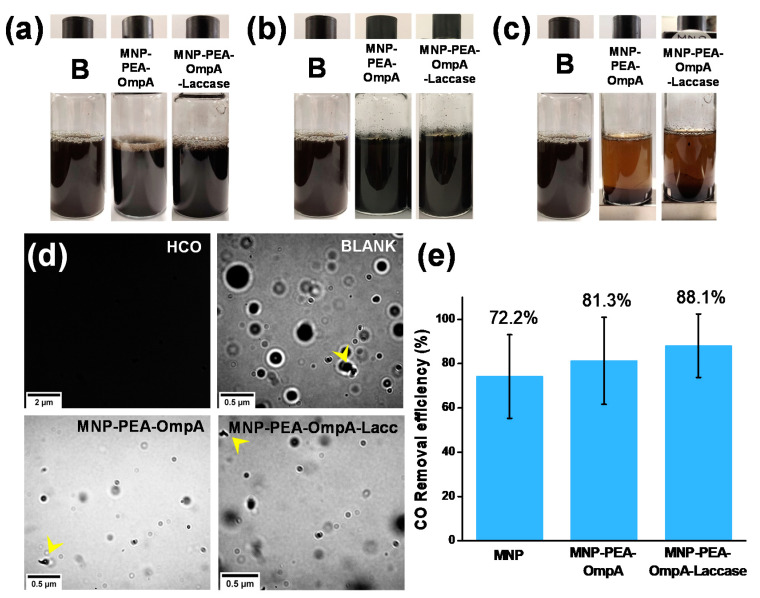
Demulsification assay of O/W emulsions by MNP-PEA-OmpA and MNP-PEA-OmpA-Laccase at 5000 ppm. Blank of O/W emulsion (B) and samples with MNP-PEA-OmpA and MNP-PEA-OmpA-Laccase (**a**) after preparation and initial exposure to treatments, (**b**) after 1 h of exposure, and (**c**) after 1 h of exposure and the application of an external magnetic field for MNP-PEA-OmpA and MNP-PREA-OmpA-Laccase bionanocompounds. (**d**) Optical microscopy images of emulsions before and after exposure to MNP-PEA-OmpA and MNP-PEA-OmpA-Laccase. (**e**) HCO removal efficiency percentage for MNP, MNP-PEA-OmpA, and MNP-PEA-OmpA-Laccase.

A representative optical microscopy image of crude oil-in-water (O/W) emulsions prepared in this work is shown in Figure 5d, BLANK. Oil drop size distribution was calculated from the images with the aid of the Image J^®^ software. Each sample was analyzed in triplicate at different z depths to find an average particle size distribution that approached 100–250 nm (not shown). Such an average size has been typically observed for miniemulsions or nano-emulsions, which generally exhibit superior stability and tolerance against sedimentation or creaming processes [85].

Figure 5a–c show images of the macroscopic changes for the O/W emulsion in the presence of the MNP-PEA-OmpA and MNP-PEA-OmpA-Laccase bionanocompounds. Figure 5a shows samples right after mixing with both bionanocompounds. This was considered the starting point and the blank for stability. Figure 5b shows images of the samples after 1 h in the presence of the bionanocompounds. At this point, a slight clarification of the water phase was observed, which could be attributed to the sedimentation of bionanocompounds. Figure 5c shows macroscopic images of the samples after the application of a magnetic field at the bottom of the glass container. The application of the field appears to help in accelerating the precipitation of dispersed solids and HCO drops. The bionanocompounds were successfully recovered after the process. Figure 5d shows microscopy images of samples treated with the bionanocompounds and exposed to the magnetic field. The number and size of drops decreased considerably with respect to the control. Suspended solids (yellow arrow) were detected in all samples, which are most likely related to the asphaltenes or resins typically present in HCO. 

Upon the recovery of the bionanocompounds from the treated samples, we measured the HCO remaining in them. Briefly, 1 mL of residual water was mixed with 3 mL of petroleum ether and sonicated in an ultrasonic bath for 5 min. The mixture was decanted to separate the polar (water) from a non-polar phase (ether). The HCO preferentially partitioned into the ether phase, which was collected and quantified spectrophotometrically in a UV-VIS instrument at 225 nm (HCO detection wavelength [15]). Extracts from O/W emulsions in the absence of treatment by the bionanocompounds were used to build a standard concentration curve (Supplementary Figure S7). Figure 5e shows the HCO removal efficiency for each treatment. The highest efficiency was achieved by the MNP-PEA-OmpA-Laccase bionanocompound, followed by the MNP-PEA-OmpA and finally the bare MNPs. This suggests that co-immobilization could lead to a synergistic interaction of proteins and HCO. Further evidence will be needed to support such a notion.

MNPs have the potential to enable numerous industry applications as their exceptional surface area and amenability for functionalization facilitate coupling of molecules with an ample range of functionalities. One of such applications is the separation of oil-water emulsions where the MNPs increase the density of the water present in the emulsion, thereby accelerating the rate of water settling. Additionally, cationic surfactant-coated MNPs have demonstrated that oil droplets are successfully separated from the negatively charged oil-in-water emulsions. This is in contrast with anionic surfactant-coated MNPs that showed no interfacial activity, thereby indicating that the stable adsorption of MNPs at the water/oil interface caused by electrostatic attractions plays a vital part in effective separation [42,86]. Additionally, several studies have found that the demulsifying effect in O/W emulsions is enabled by the coalescence of droplets in the presence of a magnetic field. This is explained by the presence of the interfacially active MNPs at the interface capable of forming Pickering emulsions and subsequently promoting droplet accumulation by the influence of the magnetic force [77]. We hypothesize, therefore, that the separation yield may be increased by adding MNP-PEA-OmpA and MNP-OmpA-Laccase at higher concentrations, as has been previously shown elsewhere [15,40,83]. Dosages of the developed nanocompounds above 0.3 wt% showed higher HCO removal efficiencies.

### 3.7. Gas Chromatography-Mass Spectrometry (GC-MS) Characterization of Residual Water

The treated O/W emulsions were also analyzed by GC–MS analysis to semi-quantitatively estimate changes in the composition of HCO with respect to the blank—i.e., untreated—O/W emulsion. Chromatograms of crude oil are shown in Supplementary Figure S8. Due to the complexity of crude oil, only peaks with the higher intensities (most abundant) were taken as a reference to estimate the changes. Supplementary Figure S8a shows the chromatogram for the blank, which presents three representative peaks at the retention times 21.9, 23.3, and 24.6 s (labeled as 9, 10, and 12). These peaks likely correspond to heptadecane, octadecane, and nonadecane, respectively. After treatment with the bionanocompounds, the intensities of these peaks declined, which is most likely due to their degradation. This was also the case of peaks 1, 13, and 15, which correspond to decamethylcyclopentasiloxane, methyl 14-methylpentadecanoate, and methyl cis-9-octadecenoate, respectively (Supplementary Figure S8b,c).

The relative areas of peaks for compounds in the treated samples were estimated with respect to the highest intensity peak in the blank (i.e., pentadecane, retention time 19.1146 s). Table 2 summarizes the peaks where major changes in composition were observed, as evidenced by a reduction in the area under the curve. Additionally, we estimated the removal efficiencies (RE(%)) for the relevant compounds by following the relation reported by Parthipan et al. [24]: RE (%) = 100 − (As × 100/Ac), where As is the total area of peaks in every sample and Ac is the total area of peaks in the reference sample. Except for nonadecane, the removal efficiencies of the involved compounds were the highest for the MNP-PEA-OmpA-Laccase bionanocompound. This confirms that the designed system was not only capable of separating the emulsions but of biodegrading some of the most abundant compounds of HCO. Even though the semi-quantitative analysis presented here allowed us to confirm the potential of our bionanocompounds, a more robust chromatography analysis with the aid of internal standards will be useful for determining more precisely the removal efficiencies of the least abundant compounds.

## 4. Conclusions

Particulate materials have played a fundamental role in the oil and gas industry due to their ability to stabilize or destabilize colloidal systems. As a result, since the beginning of the century they have found several applications ranging from the formulation of drilling fluids to the treatment of effluents [37]. With the increase in the popularity of nanomaterials, the past two decades have seen their incursion in this industry in an attempt to enhance the efficiencies of numerous conventional processes [58,87,88,89]. For instance, different types of nanomaterials have been implemented for the treatment and recovery of emulsified effluents with moderate efficiencies [77,90]. In parallel, alternative remediation processes such as those based on biosurfactants have gained attention due to the possibility of operating at milder conditions [91,92]. In the same line, the removal of environmentally concerning compounds present in effluents has been addressed with the aid of enzymes and particularly Laccases. Here, we aimed at combining the potential of these technologies by synthesizing bionanocompounds based on MNPs, the biosurfactant protein OmpA, and the enzyme Laccase. The synthesis proceeded by the functionalization of MNPs with the polymeric surface spacer PEA, followed by their independent immobilization and the co-immobilization of the two proteins to obtain the MNP-PEA-OmpA, MNP-PEA-Laccase, and MNP-PEA-OmpA-Laccase bionanocompounds.

We synthesized the MNPs by chemical co-precipitation and confirmed a diameter of about 80 nm by DLS and TEM. Functionalization and protein immobilization were successfully confirmed via FTIR and TGA. The estimated efficiency of total protein immobilization approached about 2.5%, 5.0%, and 5.5% for the MNP-PEA-OmpA, MNP-PEA-Laccase, and MNP-PEA-OmpA-Laccase bionanocompounds. These results suggested that immobilization took place under possible competing protein–protein and protein–surface interactions. The immobilization yield for the Laccase alone after co-immobilization (MNP-PEA-OmpA-Laccase) was estimated by a colorimetric assay based on ABTS to be >97%.

Demulsification assays were conducted for O/W 1 wt% emulsions. Separation was achieved at 5000 ppm of the MNP-PEA-OmpA and MNP-PEA-OmpA-Laccase bionanocompounds. This was further improved by applying an external magnetic field to reach HCO removal efficiencies of 81% and 88%, respectively. These experiments demonstrated that the bionanocompounds can be recovered after treatment, which opens the possibility for potentially recycling them back into the process. The demulsifier activity of the recovered bionanocompounds needs to be assessed in detail.

The potential of the bionanocompounds in the bioremediation of emulsified effluents was tested by measuring changes in the composition of the selected compounds. The results indicate that, in the presence of the MNP-PEA-OmpA and MNP-PEA-OmpA-Laccase bionanocompounds, we observed degradation efficiencies in the range of 5% to 50%. The highest degradation efficiencies were for heptadecane and hexadecane 2,6,10,14-tetramethyl, which approached 25% and 50%, respectively. For future studies, we recommend the use of internal standards for a more quantitative determination. Overall, our results hold significant promise for the simultaneous separation and treatment of emulsified effluents from the oil and gas industry. Moreover, due to the remaining magnetic susceptibility, chances are high that they can be incorporated into a separation/treatment unit operating cyclically.

## Figures and Tables

**Table 1:** Ion content in brine.

Ion	Concentration [ppm]
	Brine with All Ions
CL^−^	62,819
Na^+^	35,393
Ca^2+^	3253
Mg^2+^	909
HCO^3−^	218
SO_4_^2−^	49

**Table 2:** Changes in composition for the selected compounds present in O/W samples after treatment with the bionanocompounds.

			Relative Area		Removal Efficiency	
Peak N°	Compound	Blank	MNP-PEA-OmpA	MNP-PEA-OmpA-Laccase	MNP-PEA-OmpA	MNP-PEA-OmpA-Laccase
5	Pentadecane	100.00	97.43	94.44	2.57	5.56
7	Naphthalene, 1,6,7-trimethyl	34.91	33.77	31.84	3.28	8.79
9	Heptadecane	73.24	58.18	53.25	20.56	27.30
10	Octadecane	61.85	66.01	53.80	0.00	13.01
11	Hexadecane, 2,6,10,14-tetramethyl	48.41	25.31	23.90	47.71	50.62
12	Nonadecane	53.35	50.12	63.84	6.04	0.00
14	Eicosane	53.17	49.18	44.04	7.51	17.17

## Supplementary Materials

The following are available online at https://www.mdpi.com/2079-4991/10/11/2278/s1. Figure S1: BSA standard concentration curve. Figure S2: Preparation protocol of CO/W stable emulsions. A. Heating of brine and crude oil phases to 60 °C. B. Mixer positioning where the Da/Dt was maintained at about 1. Da corresponded to the propeller diameter and Dt that of the vessel. C. Addition of NP10 surfactant under vigorous mechanical agitation at 2000 RPM. D. Incorporation of pre-heated CO under vigorous mechanical agitation at 5000 RPM. The mixing process was carried out for 15 min. Figure S3: Second derivative of free OmpA (green) and MNP-OmpA (magenta) in the region of Amide I (1600 to 1700 cm^−1^). Figure S4: Remaining catalytic activity determination assay by ABTS oxidizing of samples. Figure S5: ΔBS recording of O/W emulsion in the presence of A. 200, B. 500, C. 1000 and D. 5000 ppm of free OmpA. Figure S6. ΔBS recording of O/W emulsion in the presence of A. 200, B. 500, C. 1000 and D. 5000 ppm of free MNP. Figure S7: Calibration curve for concentration of HCO in petroleum ether measured at 225 nm using petroleum ether as blank. Samples were prepared in serial dilutions from 10 μg/mL. High content samples were disregarded from the curve due to oversaturation of the detector in the instrument. Figure S8: GC-MS chromatogram for treatments with A. Blank B. MNP-OmpA and C. MNP-OmpA-Laccase. Table S1: General Data and Values. Table S2. Most significant peaks identified.
