# Supplementary material for: Novel Bionanocompounds: Outer Membrane Protein A and Laccase Co-Immobilized on Magnetite Nanoparticles for Produced Water Treatment
Source: Nanomaterials (Basel). 2020 Nov 17;10(11):2278. doi: 10.3390/nano10112278 (PMC7698600; doi:10.3390/nano10112278)
Supplement: Supplementary file 1 [file nanomaterials-10-02278-s001.pdf]

nanomaterials

Supplementary Materials

Novel Bionanocompounds: Outer Membrane Protein A and Laccase Co-Immobilized on Magnetite Nanoparticles for Produced Water Treatment

Nathaly Rangel-Muñoz [1], Andres Fernando González-Barrios [2], Diego Pradilla [2], Johann F. Osma [3] and Juan C. Cruz [1,4,*]

[1] Department of Biomedical Engineering, Universidad de Los Andes, Carrera 1 este No 19A-40, Bogotá 111711, Colombia; in.rangel@uniandes.edu.co
[2] Grupo de Diseño de Productos y Procesos (GDPP), Department of Chemical and Food Engineering, Universidad de los Andes, Carrera. 1 este No. 19a–40, Bogotá 111711, Colombia; andgonza@uniandes.edu.co (A.F.G.-B.); d-pradil@uniandes.edu.co (D.P.)
[3] CMUA, Department of Electrical and Electronic Engineering, Universidad de Los Andes, Carrera. 1 este No. 19a–40, Bogotá 111711, Colombia; jf.osma43@uniandes.edu.co
[4] School of Chemical Engineering and Advanced Materials, The University of Adelaide, Adelaide 5005, Australia
* Correspondence: jc.cruz@uniandes.edu.co

SI1. Determination of OmpA and Laccase concentration.

BSA standard curve was measured with a BSA kit in triplicate. The concentration of OmpA and Laccase was determined by this technique before immobilization in order to relate the catalytic activity (U) of Laccase with the enzyme concentration per mL.

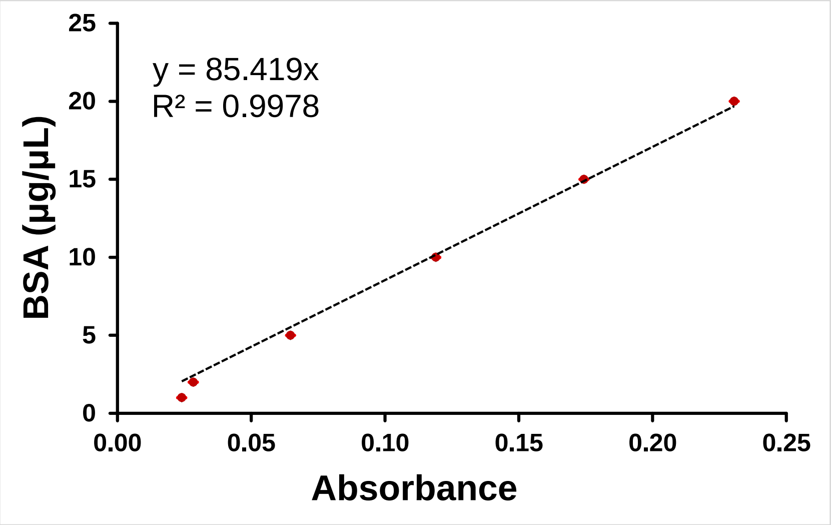

Figure S1. BSA standard concentration curve.

SI2. Calculation of available NH_2 according to MNP size.

Table S1. General Data and Values.

**MNPs**	
D [mm]	0.001*
ϱ [g/cm³]	5.18
W [g]	0.1
**APTES**	
Superficial area (Sa) [mm²]	5.37×10^{-13}
ϱ [g/mL]	0.946
MW [g/mol]	221.37
Purity (%)	99
**Glutaraldehyde**	
Superficial area (Sa) [mm²]	3.41×10^{-13}
ϱ [g/mL]	1.062
MW [g/mol]	100.117
Purity [%]	25
**PEA**	
ϱ [g/mL]	1.017
MW [g/mol]	~597.831
**OmpA**	
MW [g/mol]	35000
Purity [%]	94·:
**Laccase**	
MW [g/mol]	64000
$C_{Laccase}$ [mg/mL]·:	896
Catalytic activity [U/mL]	2000
Excess factor (ef)	2

* Diameter of MNP is assumed as 1μm in order to consider the formation of clusters of MNP during functionalization. ·: Determined by the BSA standard curve of SI1.

Amount of MNP = 100 mg

Spherical MNP is assumed:

$$A_{MNP} = 4\pi r^2 \qquad A_{MNP} = 4\pi \left(\frac{0.001}{2}\right)^2 = 3.14 \times 10^{-6}\, mm^2,$$

$$V_{MNP} = \frac{4}{3}\pi r^3 \qquad V_{MNP} = \frac{4}{3}\pi \left(\frac{0.001}{2}\right)^2 = 5.23 \times 10^{-10}\, mm^3,$$

$$W_{MNP} = 5.18 \frac{g}{cm^3} \times \frac{1cm^3}{(10mm)^3} \times 5.23 \times 10^{-10}\, mm^3 = 2.7110^{-12} g.$$

APTES amount calculations (spherical molecule is assumed):

- Number of molecules (N_{molec}) in one particle:

$$N_{molec} = \frac{A_{MNP}}{Sa_{APTES}} = \frac{3.14 \times 10^{-6}\ mm^2}{5.37 \times 10^{-13} mm^2} = 5849162.011, = 5.84 \times 10^6 APTES\ molecules$$

$$N_{MNP\ mol} = \frac{N_{MNP} \times N_{molec}}{6.02{\times}10^{23}\frac{molec}{mol}},$$

$$N_{MNP\ mol} = \frac{3.68{\times}10^{10}\ MNPs \times 5.84x10^6\ APTES\ molecules}{6.02{\times}10^{23}\frac{molec}{mol}} = 3.58 \times 10^{-7}\ MNP\ mol.$$

Under theorical calculations, there will be 5.84 × 10^6 available NH_3 sites of APTES for functionalization. After including the excess of APTES, GA, and PEA and the same amount of COOH sites of the carboxyl end of oxidized PEA, that is the number of molecules of proteins that need to be added to saturate the active functionalization sites.

- Number of molecules per MNP particle ($N_{molecules}$):

$$N_{molecules} = 5.84 \times 10^6\ .$$

Amount of MNP to functionalize is 1 mg (0.1g)

- Number of MNPs (N_{MNP}):

$$N_{MNP} = \frac{Amount\ of\ MNPs}{W_{MNP}},$$

$$N_{MNP} = \frac{0.1g}{2.711{\times}10^{-12}g} = 3.68 \times 10^{10}\ MNPs.$$

- Number of necessary Mol per particle ($N_{mol \times part}$):

$$N_{mol \times part} = \frac{N_{MNP} \times N_{molecules}}{6.02{\times}10^{23}\ molec/mol},$$

$$N_{mol \times part} = \frac{3.68{\times}10^{10} \times 5.84{\times}10^6}{6.02{\times}10^{23}\ molec/mol} = 3.56 \times 10^{-7}.$$

MNP-PEA-OmpA

- Mol of OmpA ($N_{OmpA\ mol}$):

$$N_{OmpA\ mol} = N_{mol \times part} = 3.56 \times 10^{-7} OmpA\ mol,$$

$$W_{OmpA} = N_{OmpA\ mol}\ \times\ MW\ OmpA$$

$$W_{OmpA} = 3.56 \times 10^{-7}\ OmpA\ mol\ \times\ 35000\frac{g}{mol} = 0.0124\ g,$$

$$W_{ompA} = \frac{0.0124\ g}{0.94} = 0.0132\ g\ \times\ \mathbf{2} =\ \sim 26.4\ mg\ OmpA\ .$$

MNP-PEA-Laccase

- Mol of Laccase ($N_{Lacc\ mol}$):

$$N_{Lacc\ mol} = N_{mol \times part} = 3.56 \times 10^{-7} Laccase\ mol,$$
$$W_{Lacc} = N_{Lacc\ mol} \times MW\ Laccase,$$

$$W_{Lacc} = 3.56 \times 10^{-7}\ Laccase\ mol \times 64000 \frac{g}{mol} = 0.0227\ g = 22.7\ mg,$$

$$V_{Lacc} = \frac{22.7\ mg}{896.6\ mg/mL} = 0.025 \times \mathbf{2} mL = \sim 50\ \mu L\ Laccase,$$

$$U_{Laccase} = 2000 \frac{U}{mL} \times 0.05\ mL = \sim 100\ U.$$

MNP-PEA-OmpA-Laccase

$$N_{OmpA\ mol} = \frac{1}{2} N_{mol \times part} = 1.78 \times 10^{-7} OmpA\ mol,$$

$$W_{OmpA} = N_{OmpA\ mol} \times MW\ OmpA,$$
$$W_{OmpA} = 1.75 \times 10^{-7}\ OmpA\ mol \times 35000 \frac{g}{mol} = 0.0062\ g,$$

$$W_{ompA} = \frac{0.0062\ g}{0.94} = 0.0065\ g \times \mathbf{2} = \sim 13\ mg\ OmpA,$$

$$N_{Lacc\ mol} = N_{MNP\ mol} = 1.78 \times 10^{-7} Laccase\ mol,$$
$$W_{Lacc} = N_{Lacc\ mol} \times MW\ Laccase$$
$$W_{Lacc} = 1.78 \times 10^{-7}\ Laccase\ mol \times 64000 \frac{g}{mol} = 0.0113\ g = 11.3\ mg,$$
$$V_{Lacc} = \frac{11.3\ mg}{896.6\ mg/mL} = 0.012\ mL \times \mathbf{2} = \sim 25\ \mu L\ Laccase,$$

$$U_{Laccase} = 2000 \frac{U}{mL} \times 0.025\ mL = \sim 50\ U.$$

SI3: Preparation of Crude Oil-in-Water emulsion procedure.

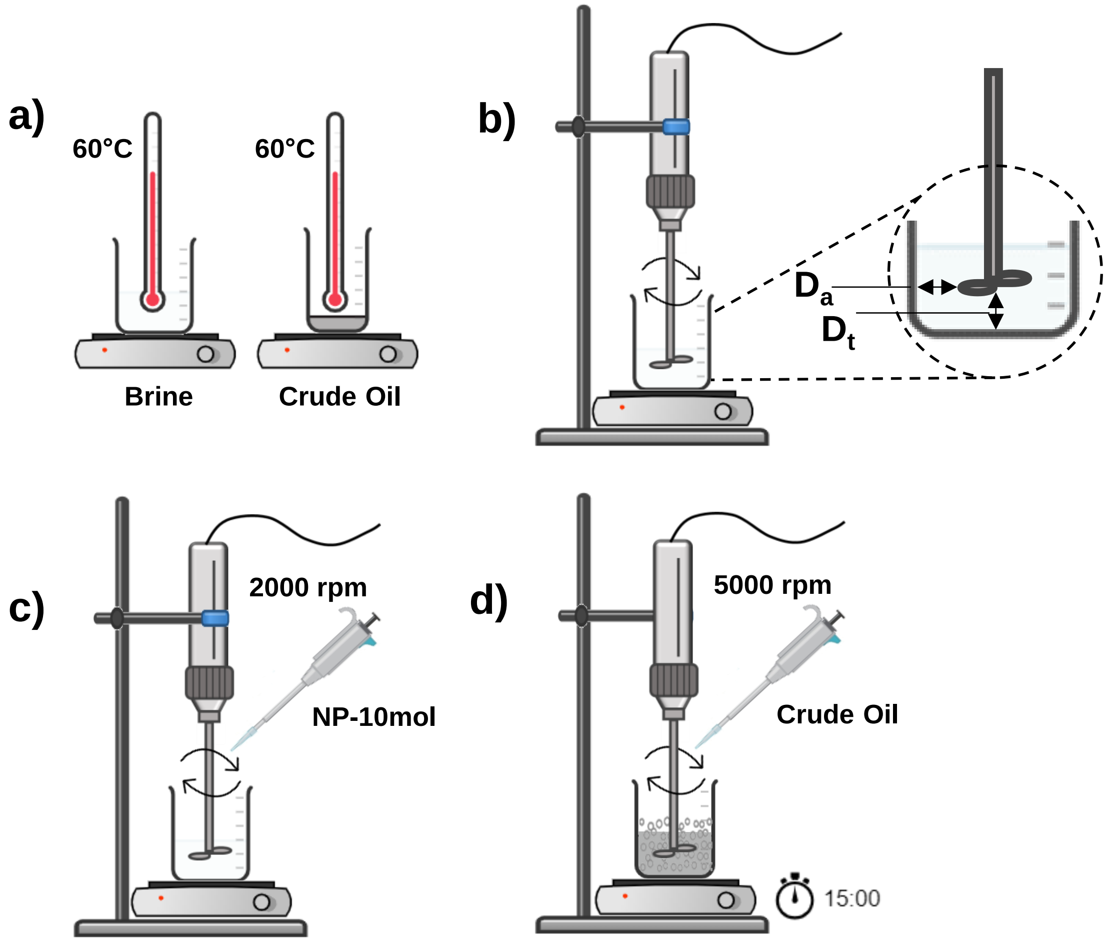

Figure S2. Preparation protocol of CO/W stable emulsions. (**a**) Heating of brine and crude oil phases to 60ºC. (**b**) Mixer positioning where the Da/Dt was maintained at about 1. Da corresponded to the propeller diameter and Dt that of the vessel. (**c**) Addition of NP10 surfactant under vigorous mechanical agitation at 2000 RPM. (**d**) Incorporation of pre-heated CO under vigorous mechanical agitation at 5000 RPM. The mixing process was carried out for 15 min.

SI4. Second derivative study of free OmpA and immobilized OmpA in MNP-OmpA bio-nanocompound.

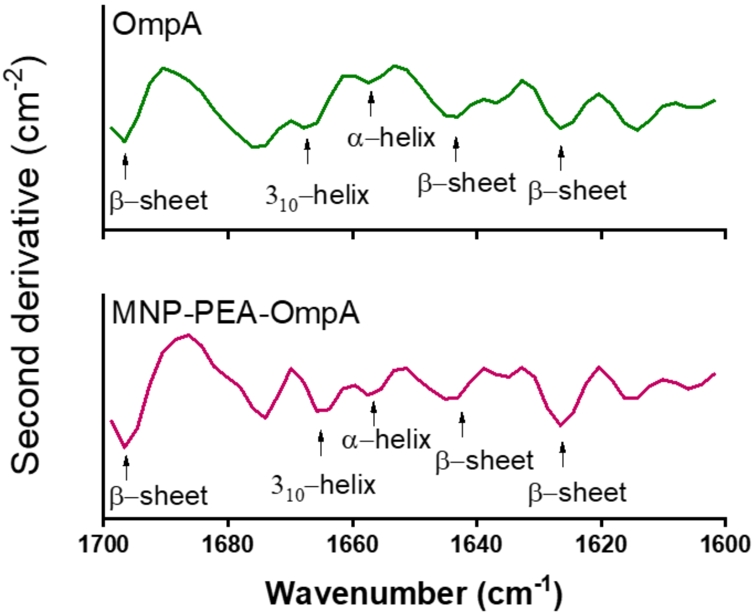

Figure S3. Second derivative of free OmpA (green) and MNP-OmpA (magenta) in the region of Amide I (1600 to 1700 cm^{-1}).

SI5. Enzymatic activity determination of immobilized Laccase*.

For determining the enzymatic activity of samples, we used the Lambert–Beer equation, where the concentration C is in molar units $[M]$, Δt, in the time interval, $\frac{\Delta ABS}{\Delta t}$, in the change in absorbance in a time interval of 1 minute. d is the long light direction (1 cm) and ε is the extinction coefficient of the substance. In the case of ABTS, it is 29,300 M^{-1} at a 436 nm wavelength. The final volume of samples is of 1500 μL. Additionally, U represents the units of Enzymatic activity (Ea) that correspond to the oxidizing of 1 μmol ABTS/min.

$$Enzymatic\ Activity(Ea)\left[\frac{U}{L}\right]=\frac{\Delta C}{\Delta t}=\left(\frac{\frac{\Delta ABS}{\Delta t}}{\varepsilon\,.\,d}\right).(dilution\ factor)$$

$$Ea\left[\frac{U}{L}\right]=\left(\frac{x_{ABS}\cdot\frac{1}{min}}{29300\ M^{-1}cm^{-1}.1\ cm}\right).\left(\frac{1500\ \mu L}{Vol_{sample}\ \mu L}\right),$$

$$Ea\left[\frac{U}{L}\right]=\left(\frac{1500}{29300}\right)\left(\frac{x_{ABS}}{Vol_{sample}}\right).\left(\frac{M}{min}\right),$$

$$Ea\left[\frac{U}{L}\right]=\left(\frac{1500}{29300}\right)\left(\frac{x_{ABS}}{Vol_{sample}}\right).\frac{mol\left(\frac{1\times10^{6}\mu mol}{mol}\right)}{min.L},$$

$$Ea\left[\frac{U}{L}\right]=51194.54\left(\frac{x_{ABS}}{Vol_{sample}}\right).\frac{U}{L},$$

$$Ea\left[\frac{U}{mL}\right]=51194.54\left(\frac{x_{ABS}}{Vol_{sample}}\right).\frac{U}{L\left(\frac{1000mL}{L}\right)},$$

$$Ea\left[\frac{U}{mL}\right]=51.19\left(\frac{x_{ABS}}{Vol_{sample(\mu L)}}\right).\frac{U}{mL}.$$

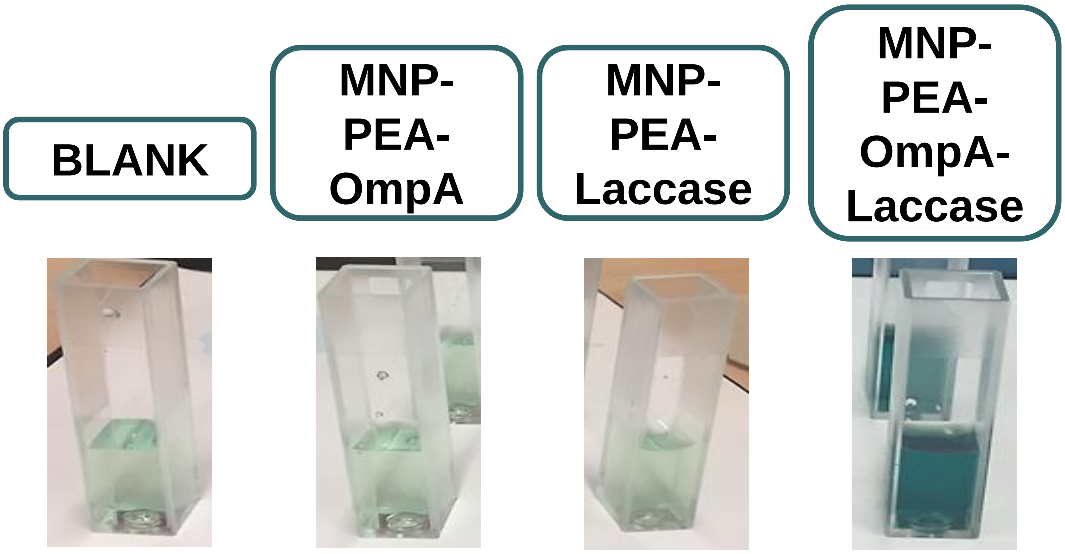

Figure S4. Remaining catalytic activity determination assay by the ABTS oxidizing of samples.

SI6. Turbiscan assay of destabilization of O/W emulsion with free OmpA

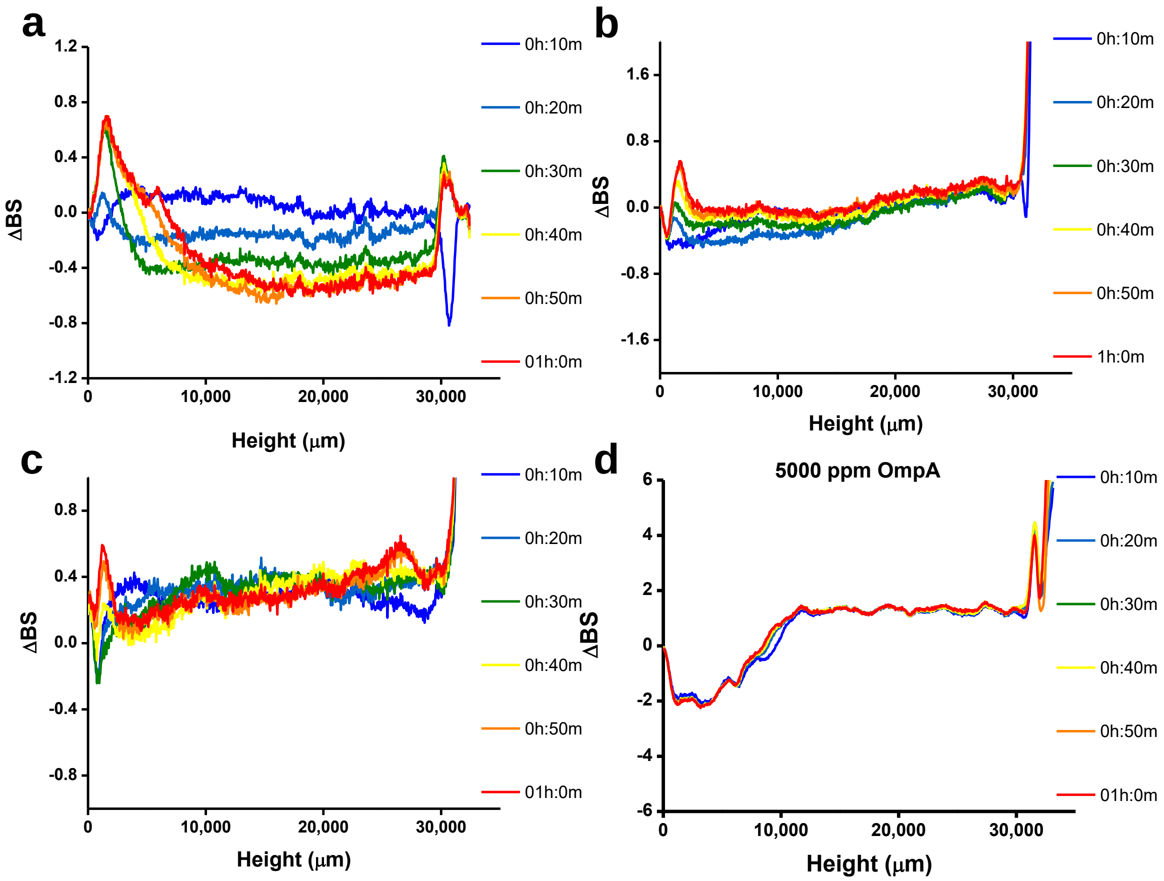

Figure S5. ΔBS recording of O/W emulsion in the presence of (**a**) 200, (**b**) 500, (**c**) 1000, and (**d**) 5000 ppm of free OmpA.

SI7. Turbiscan assay of destabilization of O/W emulsion with free OmpA

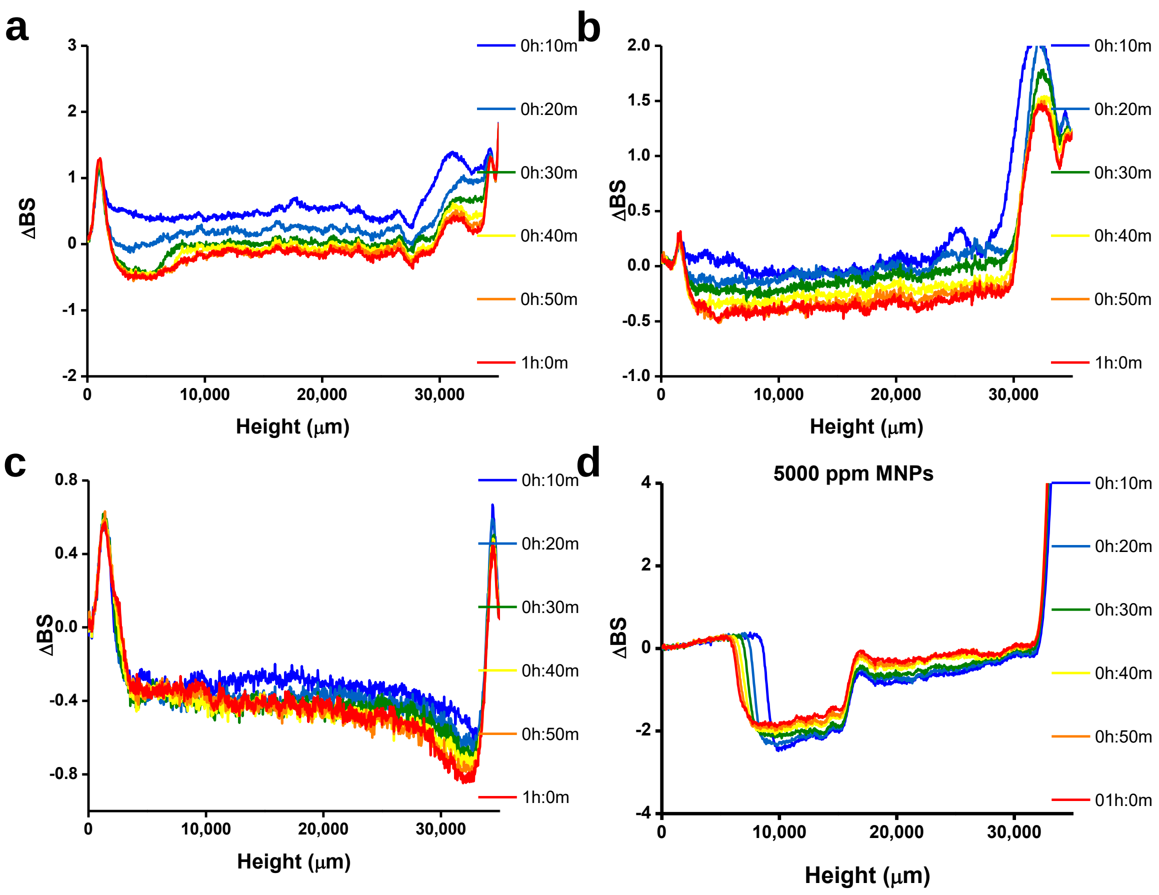

Figure S6. ΔBS recording of O/W emulsion in the presence of (**a**) 200, (**b**) 500, (**c**) 1000, and (**d**) 5000 ppm of free MNP.

SI8. Standard concentration curve of HCO

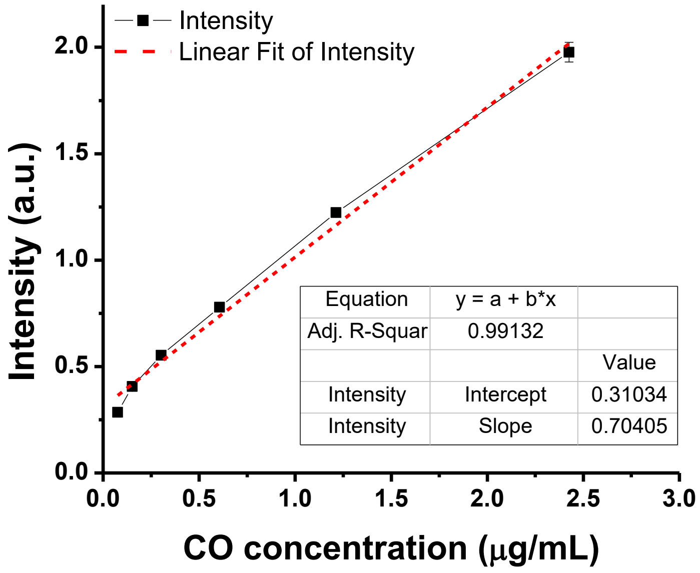

Figure S7. Calibration curve for the concentration of HCO in petroleum ether measured at 225 nm using petroleum ether as blank. Samples were prepared in serial dilutions from 10 μg/mL. High-content samples were disregarded from the curve due to the oversaturation of the detector in the instrument.

SI9: GC-MS chromatography analysis

Table S2. Most significant peaks identified.

# Peak	Retention time [s]	Compound
1	12.9829	Cyclopentasiloxane, decamethyl
2	15.8856	Tridecane
3	17.5526	Tetradecane
4	17.7799	Naphthalene, 2,6-dimethyl-
5	19.1146	Pentadecane
6	19.5401	Carbonic acid, nonyl phenyl ester
7	20.2454	Naphthalene, 1,6,7-trimethyl-
8	20.5951	Hexadecane
9	21.994	Heptadecane
10	23.3287	Octadecane
11	23.4104	Hexadecane, 2,6,10,14-tetramethyl
12	24.5936	Nonadecane
13	24.885	Pentadecanoic acid, 14-methyl-, methyl ester
14	25.8059	Eicosane
15	26.9192	9-Octadecenoic acid (Z)-, methyl ester
16	26.9658	Heneicosane
17	28.0791	Docosane

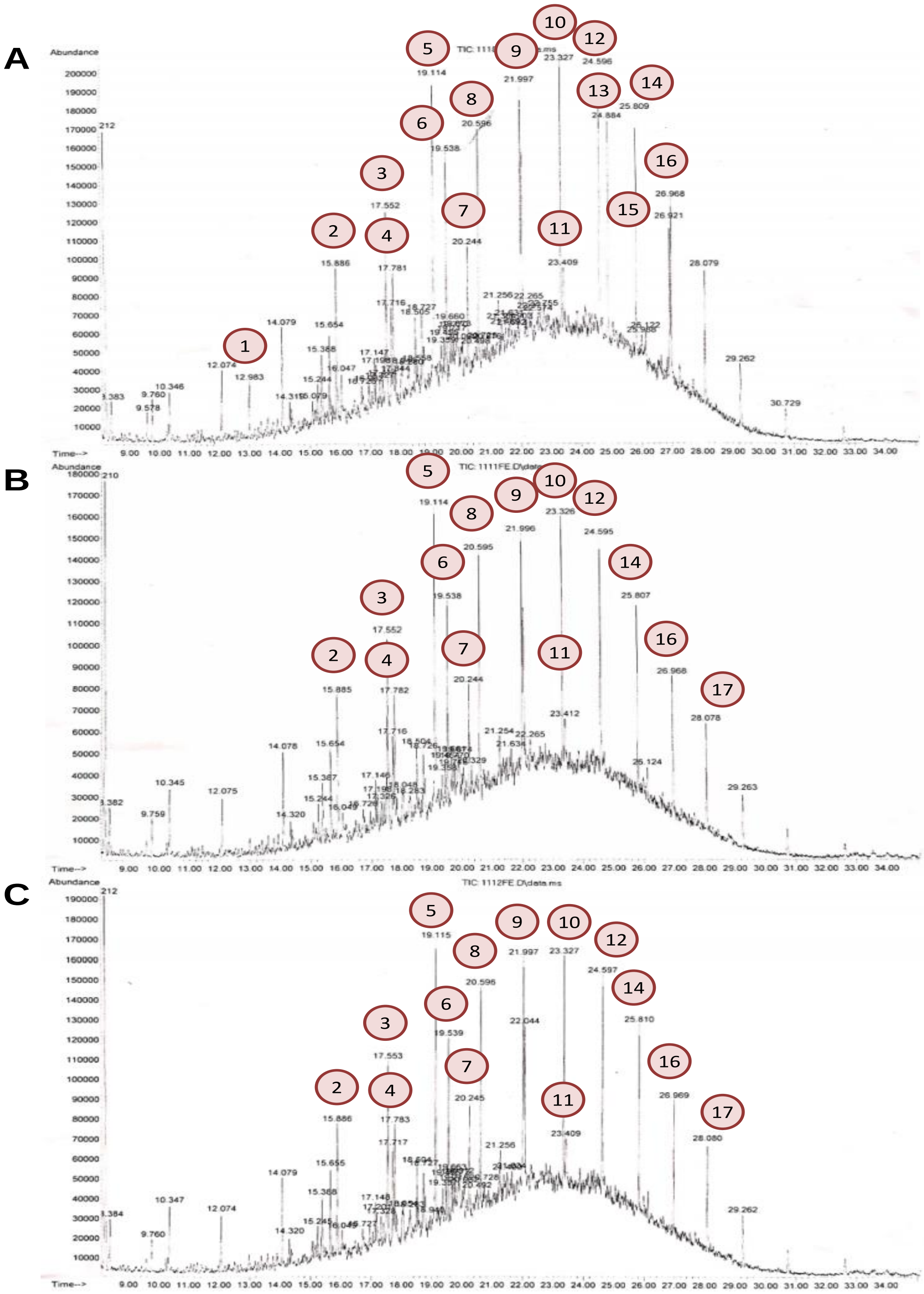

Figure S8. GC-MS chromatogram for treatments with (**A**) Blank, (**B**) MNP-OmpA, and (**C**) MNP-OmpA-Laccase.

© 2020 by the authors. Licensee MDPI, Basel, Switzerland. This article is an open access article distributed under the terms and conditions of the Creative Commons Attribution (CC BY) license (http://creativecommons.org/licenses/by/4.0/).
